# UV-Induced Cell Death in Plants

**DOI:** 10.3390/ijms14011608

**Published:** 2013-01-14

**Authors:** Ganesh M. Nawkar, Punyakishore Maibam, Jung Hoon Park, Vaidurya Pratap Sahi, Sang Yeol Lee, Chang Ho Kang

**Affiliations:** Division of Applied Life Sciences (BK21 program) and Plant Molecular Biology and Biotechnology Research Center, Gyeongsang National University, Jinju 660-701, Korea; E-Mails: ganeshtnau@gmail.com (G.M.N.); punya.maibam@gmail.com (P.M.); jazzc@nate.com (J.H.P.); vpsahi@hotmail.com (V.P.S.)

**Keywords:** UV radiation, reactive oxygen species (ROS), cell death

## Abstract

Plants are photosynthetic organisms that depend on sunlight for energy. Plants respond to light through different photoreceptors and show photomorphogenic development. Apart from Photosynthetically Active Radiation (PAR; 400–700 nm), plants are exposed to UV light, which is comprised of UV-C (below 280 nm), UV-B (280–320 nm) and UV-A (320–390 nm). The atmospheric ozone layer protects UV-C radiation from reaching earth while the UVR8 protein acts as a receptor for UV-B radiation. Low levels of UV-B exposure initiate signaling through UVR8 and induce secondary metabolite genes involved in protection against UV while higher dosages are very detrimental to plants. It has also been reported that genes involved in MAPK cascade help the plant in providing tolerance against UV radiation. The important targets of UV radiation in plant cells are DNA, lipids and proteins and also vital processes such as photosynthesis. Recent studies showed that, in response to UV radiation, mitochondria and chloroplasts produce a reactive oxygen species (ROS). *Arabidopsis metacaspase-8* (*AtMC8*) is induced in response to oxidative stress caused by ROS, which acts downstream of the *radical induced cell death* (*AtRCD1*) gene making plants vulnerable to cell death. The studies on salicylic and jasmonic acid signaling mutants revealed that SA and JA regulate the ROS level and antagonize ROS mediated cell death. Recently, molecular studies have revealed genes involved in response to UV exposure, with respect to programmed cell death (PCD).

## 1. Introduction

Light energy from the sun and the ability of photoautotrophs to fix that energy in the presence of chlorophyll has promoted life on earth. Apart from being an energy source, light acts as a major environmental cue for plants to adjust to the surrounding conditions. In nature, solar radiation comprises different wavelengths of electromagnetic radiation and is broadly classified as Ultraviolet Radiation (UV < 400 nm), Photosynthetically Active Radiation (PAR~400–700 nm) and Far Red Radiation (FR~700–780 nm). Approximately 7%–9% of the total solar radiation reaching the earth’s surface is in the UV range (200–400 nm) [1–4]. UV radiation is broadly classified based on wavelength as UV-C radiation (200–280 nm), which is the most hazardous range of UV light but physiologically insignificant since these wavelengths are completely absorbed by the atmosphere; UV-B radiation (280–320 nm), which is filtered through the stratospheric ozone layer and, therefore, only a small proportion reaches the earth surface; and UV-A radiation (320–400 nm), which cannot be absorbed by the ozone layer and is fully transmitted to the earth’s surface. Among these three types of UV radiation, UV-B is of prime importance despite its small proportion *i.e*., 1.5% of total radiation, because of its severe damaging effects on plant growth and development. UV-A radiation, although it represents 6.3% of total radiation, is less harmful than the other wavelengths of UV radiation [3].

The flux of solar radiation is strongly modified when transmitted through the earth’s atmosphere and it greatly varies due to different factors like solar angle (time of the day, season, latitude), altitude, stratospheric ozone, cloud cover and shading [5, 6]. The ozone layer acts as a key component protecting living beings from the damaging UV radiation. Anthropogenic factors, such as the release of chlorofluorocarbons into the atmosphere, can result in a depletion of the ozone layer of ~5% [7]. In general, a 1% reduction in the ozone layer results in a 1.3%–1.8% increase in the amount of biologically active UV-B radiation [3]. The adoption of the international Montreal Protocol, which seeks to reduce factors that deplete the ozone layer, is a possible solution to this complex problem. Despite such efforts, the global ozone level is still lower than in the 1970s and a return to that state is not expected for several decades [8]. Moreover, there has been a significant increase in UV-B radiation reaching the earth’s surface between 1979 and 2008, over all latitudes except the equatorial zone, with the largest increments taking place at mid to high latitudes in the Southern Hemisphere [9, 10]. Thus, this scenario of changing climate suggests that life on earth will be continuously exposed to higher dosages of UV radiation. It is therefore important to study in detail the effect of UV radiation on plants.

The current knowledge regarding ecophysiological impact of UV radiation on plants has come largely through field experiments using natural or moderately higher levels of UV-B radiation. The results from all these experiments can be summarized as UV-B radiation has very small (<20%) inhibitory effect on plant growth, on activation of acclimation response and on interference between the interaction of plants and herbivores [11–13]. Apart from field condition experiments, data from laboratory controlled conditions has helped us in the understanding of UV-B perception, signaling and UV induced damage. It has been documented that plants respond differentially to UV fluence rate as well as wavelength [4, 14]. Lower doses of UV-B stimulate photomorphogenesis in etiolated plants while higher doses of UV-B or UV-C result in cellular damage [4, 15]. It has been reported that UV-C overexposure can induce programmed cell death (PCD) by activation of caspase-like proteases, oligonucleosomal DNA fragmentation, and appearance of apoptotic nuclear morphology in *Arabidopsis thaliana* [16–18]. Moreover, UV-B overexposure can also induce PCD in a BY-2 tobacco cell line [19]. The current review focuses on the detailed mechanism of PCD in response to higher UV radiation and its physiological significance.

## 2. Perception and Signaling of UV Light in Plants

Light regulates different growth stages and vital physiological process throughout the lifecycle of plants. Owing to the importance of light, plants evolved different photoreceptors for perception of light through which they can sense the quality (wavelength), intensity, duration (including day length) and direction of light [20]. Plants have evolved complex and sophisticated transcriptional networks involving different photoreceptors that mediate developmental changes in response to light. These developmental changes, which include seed germination, de-etiolation, gravitropic orientation, plant architecture, stomatal movement and reproductive development at time of flowering, have been extensively reviewed [21–23]. In *Arabidopsis*, three classes of UV-A/blue photoreceptors *viz*., cryptochromes (CRY1, CRY2 and CRY3), phototropins (PHOT1, PHOT2) and members of the Zeitlupe family (ZTL, FKF1, and LKP2) [24–27] have been identified, while red (R) and far-red (FR) light is maximally absorbed by the phytochromes (PHYA, PHYB, PHYC, PHYD and PHYE) [28, 29]. Different studies have shown that UV-B radiation evokes diverse phenotypic responses, including hypocotyl growth inhibition, cotyledon expansion, phototropic curvature and induction of UV-B-protecting pigmentation, in plants [15, 30–34]. In the last decade, whole genome expression profiling has identified specific pathways activated in response to low-level UV-B [35]. These photomorphogenic UV-B responses have been shown to be mediated through UV RESISTANCE LOCUS8 (UVR8) protein [20,34, 36] which has been characterized as a UV-B photoreceptor [37–39]. UVR8 is a β-propeller protein showing similarity with human guanine nucleotide exchange factor *Regulator of Chromatin Condensation 1 (RCC1)* and its crystal structure is maintained by salt bridge interactions between charged amino acids Arg, Asp and Glu at the dimeric interaction surface [38]. Four tryptophans (W94, W233, W285, and W337) located adjacent to salt-bridge amino acids are arranged to form two pyramids across the dimeric interface [38]. Absorption of UV-B light by one or more tryptophans results in a loss of excitation coupling and leads to disruption of salt-bridges and hence monomerization. Unlike other photoreceptors UVR8 lacks a specific chromophore or rather intrinsic tryptophans W285 and W233 which are important for UV-B absorbance [38,39].

**Figure 1:**
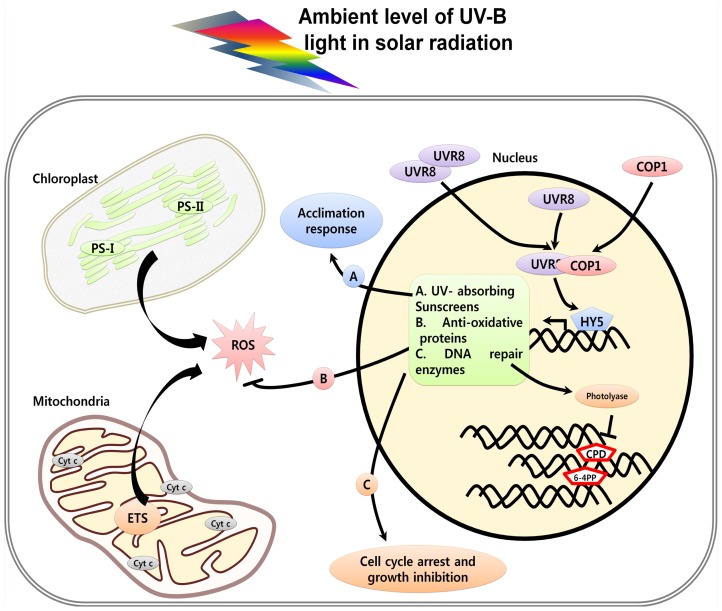
Low-fluence UV radiation activates UVR8 dependent photomorphogenesis. A, Increased level of UV-absorbing sunscreens gives acclimation response; B, Increased anti-oxidative proteins can act as ROS scavengers; C, Increased level of DNA repair enzymes can act on CPDs and 6-6 PPs lesions and may result in cell cycle arrest and overall growth inhibition.

Interestingly, the UV-B specific pathway is devoid of known light photoreceptors but involves two known central components of light signaling, *viz.*, ELONGATED HYPOCOTYL (HY5) and CONSTITUTIVE PHOTOMORPHOGENESIS 1 (COP1). The UVR8 interacts with COP1 in UV-B dependant manner to activate expression of a set of genes that protects against potential damage by UV-B exposure, including genes encoding flavonoid biosynthesis enzymes, DNA repair enzymes, and also proteins involved in mitigating oxidative stress as shown in Figure 1 [34]. The downstream components in the UVR8-COP1-HY5 pathways are summarized in Table 1.

The importance of UVR8 mediated response in the acclimatization of plants against UV-B radiation is well known from the fact that the *Arabidopsis* mutant does not accumulate flavanoids and other phenolic compounds showing that they are UV sensitive. It was reported that flavanoids also possess free-radical scavenging activity that might offer additional cellular protection [40]. Apart from induction of UV absorbing pigments, there is upregulation of DNA repair machineries such as photolyase genes. The summary of genes impaired in UV sunscreen pigments causing hyper or hypo- sensitivity to UV radiation is given in Table 2.

**Figure 2:**
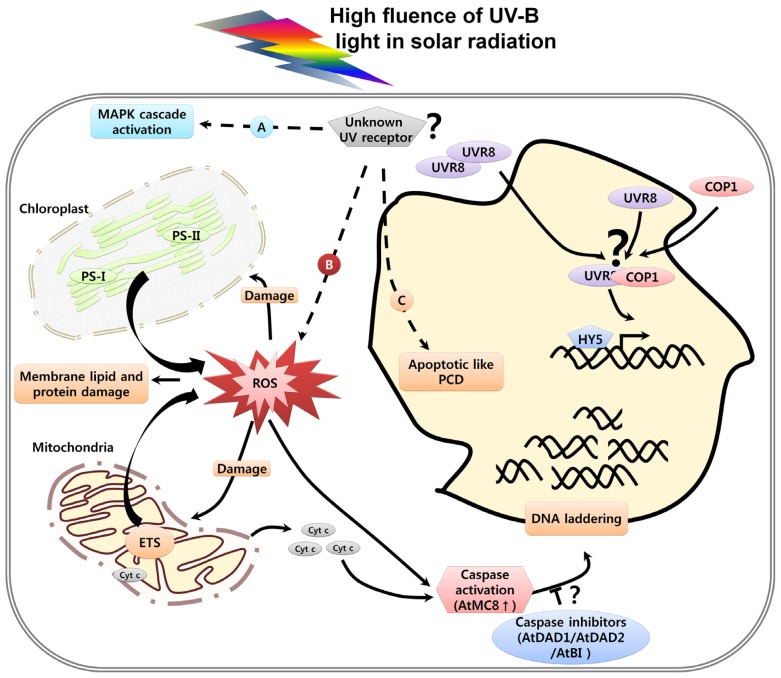
High-fluence UV radiation activates UVR8 independent stress pathway and cell death. A, Stress from UV exposure activates the mitogen-activated protein kinases (MAPK) cascade which leads to regulation of PCD; B, ROS released from chloroplast and mitochondria causes membrane lipid and protein oxidation, Mitochondrial Transmembrane Potential (MTP) loss from mitochondria results in cytochrome c release and activation of caspases and finally DNA laddering; C, High UV stress leads to A and B, which result in cell shrinkage, nuclear condensation showing Apoptotic like-PCD (AL-PCD). * Dotted arrow indicates the stress response pathway activated by yet unknown UV photoreceptor; “?” indicates the unknown UV photoreceptor, obscure role of UVR8 dependant pathway under stress conditions and the mechanistic involvement of *AtDAD1*, *AtDAD2* and *AtBI* in UV induced PCD is unclear.

Although, in the last decade we have made much progress regarding the understanding of the UV-B photoreceptor and its structure in the model plant *Arabidopsis*, there are still certain points to be considered. The fluence response curve experiment with *uvr8-2* and *hy5* lines in etiolated plants does not show significant differences from the wild type suggesting the presence of some other photoreceptors in plants [50]. It has been speculated that longer wavelengths and shorter wavelengths of UV-B radiation trigger two pathways and the shorter wavelength pathway negatively interferes with the longer wavelength pathway. The longer wavelength response can be mediated by UV-B photoreceptors and the short wavelength may represent indirect effects of UV-B exposure through general cellular stress pathways or distinct photoreceptors [14, 50]. Due to the lack of well-defined phenotypes and the accompanying damage caused by UV-B it has made it difficult to determine a UV-B photoreceptor apart from UVR8 which can respond to short wavelength radiation and regulate stress response. The UV-B induced stress pathway also known as “UV response”, is conserved in yeast, mammals and plants and involves the activation of mitogen-activated protein kinases (MAPKs) [51–53]. Recently, in *Arabidopsis*, MPK3 and MPK6 have been shown to be activated in response to UV-B stress and their mis-regulation in *mkp1* mutant results in UV-B hypersensitivity phenotypes [46]. The MPK6 has been known as a positive regulator of programmed cell death (PCD) in developmental processes like senescence [54, 55] and also in biotic stresses like pathogen attacks [56]. Thus, it has been suggested that MKP1 provides protection against UV-B-induced cell death by inhibiting UV-induced MPK3 and MPK6 activities [46]. The two UV-B response pathways namely UV-B induced photomorphogenesis regulated by UVR8 and stress induced MAPK pathway have been shown to be independent of each other and coordinately determine plant UV-B tolerance (Figure 2).

## 3. UV Induced Damage and Cell Death

Recently the UV-B light specific photoreceptors were reviewed and it was explained there how low fluence UV-B induces protective response [20]. In the current review, we discuss in detail UV light perception, signaling and important UV targets in the cell with a focus on how high intensity UV induces plant stress. The response to such stress independent of the UV photoreceptor and plants can activate the cell death pathway. Programmed cell death (PCD), is a genetically controlled self destruction mechanism in all eukaryotic multi-cellular organisms, and can be induced either as part of normal development or in response to stress. PCD is also known as apoptosis and has been well studied in animal systems. Apoptosis is characterized by specific hallmarks such as cell shrinkage, nuclear condensation and fragmentation, and eventually the breakup of the cell into “apoptotic bodies” that are eventually engulfed by phagocytes [57, 58]. The progress of plant PCD is much slower but interestingly shares many conserved components with animal apoptosis. Despite the similarities between cell death pathways in plants and animals, there is no evidence for apoptotic bodies, as well as classical caspases in plant systems. Plant cell death is described as an apoptotic-like PCD (AL-PCD) [59]. AL-PCD is now accepted as a fundamental cellular process since it plays an essential role during development, under stress conditions, in the senescence process and in response to pathogen infection in plants [60, 61]. Apart from developmental stimuli, biotic stresses, such as the pathogen induced hypersensitivity response (HR) and abiotic stresses such as heat stress and high fluence UV radiation, have been shown to induce AL-PCD [16, 62– 65]. Genes involved in cell death pathway in plants in response to UV radiation are summarized in Table 3.

### 3.1. DNA Damage, Cell Cycle Arrest and Cell Death

DNA is one of the key targets for UV-induced damage in both prokaryotic and eukaryotic cells. The adverse effects of solar radiation on living organisms are mostly due to the small amount of UV-B reaching the earth’s surface that is absorbed by cellular DNA. UV-A wavelengths are less efficient in inducing DNA damage as they are not absorbed by native DNA. It has been documented that the increase in solar UV-B radiation, due to a long-term depletion of the stratospheric ozone layer, may influence the genomic stability of plant populations [69]. The different types of DNA damage in response to UV-B or UV-C radiation includes the formation of cyclobutyl pyrimidine dimers (CPDs), (6-4) photoproducts ((6-4) PPs) [70], Inter/Intra Cross Link (ICL), 8-oxoG or even DNA Double-Strand Breaks (DSBs) [71]. The formation of CPDs represents approximately 75% of the total DNA damage by UV light [70]. The plants adopt three different strategies towards minimizing the DNA damage namely plant-specific photo reactivation pathway [70], global genome repair- nucleotide excision repair (GGR-NER) pathway [71, 72] and homologous recombination [73]. The photo reactivation pathway includes different photolyases for example in *Arabidopsis*, the *UVR2* gene encodes a photolyase (PHR1) that acts only on CPDs [74] whereas the *UVR3* gene encodes a photolyase specific for 6-4 photoproducts [75]. CPDs and 6-4 PPs can also be removed in the dark through nucleotide excision repair, endonucleolytic cleavage, release of the damaged nucleotides and strand re-synthesis [72]. The genes mentioned above involved in DNA repair pathways are summarized in Table 4.

In mammals, it has been reported that DNA damage caused by UV radiation results in cell cycle arrest and apoptosis [76] but very little is known about such mechanisms in plants. The transcriptional responses of maize to UV-B radiation under field conditions indicates that UV-B might affect the cell cycle [77]. Recently, it has been shown that UV-B radiation modulates cell cycle regulatory genes involved in G1 -to- S transition using HU (Hydroxy Urea) synchronized root tips of *Arabidopsis*. The delayed induction of *CYCD3;1* transcripts under UV-B radiation results in delayed G1-to-S transition [78]. It was also confirmed that the G1-to-S arrest induced by UV-B in root tips was a consequence of DNA damage which has been shown using *uvh1* mutant impaired in removal of CPDs [78,79]. G1-to-S arrest induced by UV-B has been suggested as a protective mechanism against UV-B-induced DNA damage, which allows time for repairing DNA damages before replication. This study concludes that the DNA damage is a possible reason behind the plant growth inhibition after UV exposure. In *Arabidopsis*, there are two proteins which can acts as sensors of DNA damage, namely, ATM (ataxia-telangiectasia mutated) and ATR (ataxia-telangiectasia and Rad3-related). The role of *Arabidopsis* ATM is more prominent under gamma radiation induced DNA damage while ATR deficient plants showed hypersensitivity to UV-B radiation and exhibited altered G2-phase cell cycle checkpoints [80, 81]. Different genes involved in cell cycle regulation impairment causing UV-induced hypersensitivity response are summarized in Table 4.

The higher doses of UV-B or UV-C radiation can induce oligonucleosomal DNA fragmentation, as a physiological response, which shows nucleosomal fragments in multiples of 180 bp like a typical apoptotic DNA ladder [16, 19]. DNA laddering is an integral part of programmed cell death (PCD) in plant systems [86, 87], confirming the role of UV radiation in induction of PCD. DNA damage is quantified by using terminal deoxynucleotidyl transferase-mediated dUTP nick-end labeling reaction (TUNEL), which detects *in situ* free 3′-OH DNA breaks [16, 88]. Although the doses of UV radiation in the above mentioned studies are physiologically irrelevant, they are still important for making experimental set ups to study PCD responses in plants.

### 3.2. Proteins and Lipids Damage

Proteins and lipids are direct targets of UV-B radiation. Since proteins strongly absorb ~280 nm or higher wavelengths, UV-B can affect the aromatic amino acids such as tyrosine, phenylalanine, and tryptophan [89]. Proteins can undergo photomodification directly through photooxidation reactions or indirectly by photosensitized production of active oxygen species and free radicals. UV-B exposures lead to damage of photosynthetic machinery including RUBISCO [90]. These direct effects are often observed in high UV-B fluence and low accompanying PAR. However, the induced loss of photosynthetic activity, often observed under lab or greenhouse conditions might not translate to the fields or natural conditions [91]. UV-B damages the ribosomes by crosslinking with the cytosolic ribosomal proteins and chloroplast ribosomal proteins to RNA, thereby transiently inhibiting translation *in vivo*. Although there is a small and significant decline in photosynthesis rate, recovery is complete by the next day and also there are no changes in the levels of chlorophylls a and b, carotenoids, or total proteins [92]. Thus, despite the presence of ribosome damage and a decrease in translation, physiological parameters were not significantly affected by the UV-B treatments, demonstrating that the treatment applied was not lethal.

Plant cell membranes contain unsaturated fatty acids, which are damaged by UV-radiation in the presence of oxygen [93, 94]. The peroxidation of membrane lipids leads to breakdown of their structure and function [95, 96]. The composition of membrane lipids such as mono- and digalactosyldiglicerides (MGDGs, DGDGs) in chloroplasts may change due to UV-radiation. The concentration of the MGDGs reduced without affecting the overall DGDG concentration and the total phospholipid levels. This reduction of the MGDGs is important for maintaining the optimal membrane structure in chloroplasts for photosynthesis under UV-B radiation [97].

### 3.3. Organelle Dysfunction in Response to UV and Cell Death

In plants, chloroplast and mitochondria are the two important cell organelles which act as sites of ROS production (Figure 2). Plant chloroplasts capture light energy during photosynthesis, a process that is very sensitive to stress conditions generating ROS or oxidative damage [98, 99]. It is known that chloroplasts are involved in different cell death processes such as leaf senescence [100]. Chloroplast involvement in PCD was confirmed by ectopic expression of mammalian anti-apoptotic Bcl-2 family members, which protect transgenic tobacco plants from PCD, induced by chloroplast-targeted herbicides [101]. The chloroplast specific ROS inhibitor 3-(3,4-dichlorophenyl1)-1,1-dimethylurea (DCMU) can retard cell death induced in response to UV overexposure. Moreover, after UV treatment and under continuous light, ROS, derived from chloroplasts, may further damage adjacent mitochondria [18].

Involvement of mitochondria in programmed cell death has been extensively studied in animals. Recently, a role for mitochondria in plant PCD was reported in response to different stimuli such as ceramide, protoporphyrin IX, the HR elicitor AvrRpt2, and UV-C radiation [18, 102]. Different stress conditions cause changes of the mitochondrial transmembrane potential (MTP) releasing cytochrome c into the intermembrane mitochondrial space, which activates caspase activity and subsequently cell death [102, 103].

### 3.4. UV-Induced Caspase Activity and Cell Death

In the animal systems, apoptotic cell death is controlled by caspases (cysteine-dependent aspartate-specific proteases), which show specific protease activity upon induction by cell death [104, 105]. In plants, caspase-like activities have been demonstrated during hypersensitive response (HR) [106] or after heat shock of suspension cells [107]. However, plants do not appear to have true caspases but rather a small family of proteins that share similarities with caspase-like domains, known as metacaspases [108]. There are nine metacaspases genes in the *Arabidopsis* genome, which can be divided into two types. Type I is comprised of three genes, while the Type II subfamily consists of six genes. Among these nine metacaspases, five genes prefer arginine rather than aspartate as a P1 amino acid at the cleavage site [109–111]. It has been hypothesized that the metacaspases are the functional homologs of animal caspases in plants.

A protease, cleaving the caspase substrate Asp-Glu-Val-Asp (DEVDase activity), is induced within 30 min and peaks at 1 h by UV overexposure under light conditions [17]. Broad-range cysteine protease inhibitors did not affect the DEVDase activity. However, caspase-1 and caspase-3 inhibitors and the pan-caspase inhibitor p35 were able to block this activity. Strong, ectopic expression of *AtDAD1* and *AtDAD2*, *A. thaliana homologs of Defender against Apoptotic Death-1*, can suppress the DNA fragmentation caused by DEVDase activity and, in turn increase cell survivability under stress conditions (Figure 2). The activation of caspase-3-like protease in UV-C-induced PCD has been documented using FRET technique which is a powerful tool for monitoring key events of PCD in living cells [112]. These data confirm that the caspase-like activities observed during HR, heat stress and UV exposure function in a similar manner to animal caspases. Apart from this indirect evidence, it has been demonstrated that there is induction of *Arabidopsis metacaspase-8* (*AtMC8*), in response to oxidative stresses caused by UV-C, H_2_O_2_, or methyl viologen [67]. The induction of *AtMC8* depends on *AtRCD1* and thus, mutant defective in either *atmc8-1/2* or *rcd 1-1* lines display tolerance to cell death induced by ultraviolet light. Interestingly, the induction of *AtMC8* transcripts in response to UV treatments also depends upon light showing it to be an integral part of plant specific PCD. However, it is not clear how light promotes cell death in response to stress.

### 3.5. UV-Induced ROS and Cell Death

In plants, ROS are generated as byproducts of essential energy generating processes such as photosynthesis and respiration. Therefore, some ROS production by chloroplasts and mitochondria is an unavoidable incidence in plants [113–115]. It was demonstrated that ROS are induced in response to different biotic and abiotic stresses. Recently, in addition to being a cell damaging agent, ROS were described as secondary signaling molecules [116]. ROS-mediated signaling is a complex mechanism that depends on the nature of the individual ROS species produced, the balance between ROS producing enzymes, and the oxidation–reduction states of various antioxidants [117]. Plants show elevated levels of ROS due to disruption of metabolic activities and increased activity of membrane-localized NADPH-oxidase in response to UV radiation [118, 119]. High UV-B radiation exposure to plants causes conditions similar to oxidative stress resulting from ROS and the gene expression pattern exhibited under both conditions is similar [120]. UVR8 mediated acclimatization is important for the survival of the plant against oxidative stress caused by UV- B radiation.

The defense responses to various pathogenic attacks [121] and other unfavorable conditions fails to control excess ROS accumulation leading to oxidative stress and may lead to cell death. Recently, the role of ROS as a trigger of UV-induced cell death processes in plants was studied. ROS acts as a signaling molecule leading to the opening of the permeability transition pore (PTP) in the mitochondrial membrane, which leads to the release of cytochrome c and the generation of more ROS, causing a feedback loop that amplifies the original PCD-inducing stress signal [58, 122]. ROS-dependent release of cyrochrome c from mitochondria during heat stress in tobacco cells suggested that the mitochondria may play a similar function in PCD in both plants and animals [123]. The role of mitochondria in the early stages of PCD induced by UV-C overexposure was confirmed using a protoplast system [18]. It was reported that before the onset of cell death, there was a loss of mitochondrial transmembrane potential (MTP). Subsequently, the mitochondria were found irregularly clumped around chloroplasts or aggregates in other places within the cytoplasm culminating in a loss of cellular movement of the mitochondria. The addition of cyclosporine, a known inhibitor of mitochondrial permeability transition pores (MPTP), effectively reduced the loss of MTP and in turn repressed cell death [18].

There are also roles for ROS and phytohormones [salicylic acid (SA), jasmonic acid (JA) and ethylene] in plant PCD [124]. In plants, the exposure to UV radiation results in transcriptional activation and repression of specific sets of genes. These regulatory pathways share common components involved in oxidative stress responses to pathogen attack, ozone exposure and wounding, including the involvement of ROS, SA, JA and ethylene. In response to UV-B exposure, SA levels increase, which triggers the induction of various pathogenesis related (PR) genes. Transgenic plants expressing *NahG,* a bacterial enzyme that degrades salicylate to catechol, have significantly lower levels of SA with a concomitant reduction in the expression of *PR1* after UV-B exposure. These transgenic lines show increased sensitivity towards UV-B radiation [125, 126]. In field conditions, UV-B radiation does not induces JA levels but increases tissue sensitivity towards JA. UV-B induces the accumulation of phenolic compounds using jasmonate-dependent (polyamine conjugates) and jasmonate-independent (flavonoids and chlorogenic acid) pathways mediated by UVR8 [127, 128]. The JA insensitive mutant *jar1* is defective in the recovery from UV-B treatment indicating that JA is important for tolerance to UV radiation [127, 129]. Like SA and JA, ethylene is also induced in response to diverse external stresses such as drought, ozone exposure and UV-B [97]. Although, ethylene is not required for induction of *PR1* gene expression in response to pathogen infection, the ethylene insensitive mutant *etr1* was unable to induce *PR1* and *PDF1.2* gene expression in response to UV-B radiation [130].

## 4. Conclusions

Global warming coupled with ozone depletion has been reported to affect the growth and development of plants negatively. The amount of damage in plants may vary depending on other environmental factors like temperature, CO_2_ level, available nitrogen and altered precipitation. A breakthrough discovery in the field of UV perception and signaling was the identification of UVR8 as a photoreceptor for UV light and UV defense responses mediated by UVR8-COP1-HY5 pathway, which increases sunscreen pigments and ROS scavenging activity upon ambient levels of UV exposure. UV-B radiation induces DNA damage resulting in cell cycle arrest and may lead to cell death. The use of higher dosage of UV-B or UV-C radiation which is physiologically irrelevant can induce PCD in plants under laboratory conditions. The stress pathway activated under high fluence UV-B radiation is independent of UVR8 signaling. UV overexposure induces oxidative burst and subsequently disrupts the function of the vital organelles, chloroplast and mitochondria. Loss of mitochondrial transmembrane potential (MTP), causes the release of cytochrome c and, in turn, activates the metacaspase cascade in plants. Plant PCD is inhibited under dark conditions suggesting that light requirement is a plant specific character of PCD, but the exact role is unknown. Different anti-apoptotic genes like *AtDAD1, AtDAD2* and *AtBI* were shown to inhibit PCD but the direct evidence for their mechanistic involvement in UV specific cell death is lacking. Moreover, how the UV photoreceptor is involved in UV mediated cell death is not yet clear. In order to answer the questions raised above, it will be important to study the effect of UV signaling components of the cell death pathways under conditions of high fluence UV radiation.

## Figures and Tables

**Table 1:** List of genes involved in UV-B light perception, signaling and stress pathway.

Symbol	AGI code	Full name	Phenotype of UV-grown mutant seedlings	Reference
*UVR8*	AT5G63860	UVB-RESISTANCE 8	hypersensitive to UV-B and blocks expression of UV-B induced genes, acts as a UV-B photoreceptor	[35, 37, 41]
*COP1*	AT2G32950	CONSTITUTIVE PHOTOMORPHOGENIC 1	hypersensitive to UV-B and blocks UVR8-dependent expression of UV-B induced genes	[42]
*HY5*	AT5G11260	ELONGATED HYPOCOTYL 5	hypersensitive to UV-B and blocks UVR8-COP1 dependent expression of chalcone synthase and flavonoid synthase pathway	[14]
*HYH*	AT3G17609	HY5-HOMOLOG	hypersensitive to UV-B and showed overlapping functions with HY5 in affecting UV-B induced gene expression	[43]
*BBX24*	AT1G06040	B-BOX DOMAIN PROTEIN 24	hypersensitive to UV-B and BBX24 interacts with COP1 and antagonizes the transcriptional activity of HY5 in response to UV-B	[44]
*RUP1*	AT5G52250	REPRESSOR OF UV-B PHOTOMORPHOGENESIS 1	no obvious phenotype but acts redundantly to RUP2. The double *rup1rup2* mutant is hypersensitive to UV-B	[45]
*RUP2*	AT5G23730	REPRESSOR OF UV-B PHOTOMORPHOGENESIS 2	hypersensitive to UV-B and acts in feedback regulation of UV signaling	[45]
*MKP1*	AT1G10210	MITOGEN-ACTIVATED PROTEIN KINASE 1	mutant is hypersensitive to acute UV-B stress due to mis-regulation of MPK3 and MPK6	[46]
*MPK3*	AT3G45640	MITOGEN-ACTIVATED PROTEIN KINASE 3	mutants display increased tolerance to UV-B radiation	[46]
*MPK6*	AT2G43790	MITOGEN-ACTIVATED PROTEIN KINASE 6		

**Table 2:** List of genes showing hyper or hypo- sensitivity to UV-B light.

Symbol	AGI code	Full name	Phenotype of UV-grown mutant seedlings	Reference
*TT4*	AT5G13930	TRANSPARENT TESTA 4/ CHALCONE SYNTHASE	has reduced flavonoids and normal levels of sinapate esters, is more sensitive to UV-B	[47]
*TT5*	AT3G55120	TRANSPARENT TESTA 5/ CHALCONE ISOMERASE	has reduced levels of UV-absorptive leaf flavonoids and the monocyclic sinapic acid ester phenolic compounds, are highly sensitive to UV-B	[47]
*TT6*	AT3G51240	TRANSPARENT TESTA 6/ FLAVANONE 3 HYDROXYLASE	similar to *tt5*, mutants are highly sensitive to UV-B light damage	[47]
*PFG1*	AT2G47460	PRODUCTION OF FLAVONOL GLYCOSIDES 1	acts downstream of HY5 and overexpression is sufficient to increase UV-B tolerance	[48]
*PFG2*	AT3G62610	PRODUCTION OF FLAVONOL GLYCOSIDES 2	hypersensitive to UV-B due to low levels of flavonol compounds n response to UV-B	[48]
*HO1*	AT2G26670	HEME OXYGENASE 1	hypersensitive to UV-C due to down regulation of flavonoid and carotenoid metabolism as well as antioxidant defense mechanisms	[49]
*ULI3*	AT5G59920	UV-B LIGHT INSENSITIVE 3	mutant is hyposensitive to UV-B and affected in its hypocotyls elongation but was also impaired in CHS and PR-1gene expression after irradiation with continuous UV-B	[15]

**Table 3:** List of genes involved in cell death pathway in plants in response to UV radiation.

Symbol	AGI code	Full name	Effect on UV-induced cell death	Reference
*AtDAD1*	AT1G32210	DEFENDER AGAINST APOPTOTIC DEATH 1	overexpression can suppress the DNA fragmentation caused by DEVDase activity and retard UV-B induced cell death	[17]
*AtDAD2*	AT2G35520	DEFENDER AGAINST CELL DEATH 2 (DAD2)		
*AtBI*	AT5G47120	BAX INHIBITOR 1	anti-apoptotic protein increases cell survivability under abiotic stresses	[66]
*AtMC8*	AT1G16420	METACASPASE 8	induced in response to UV-C radiation and induces cell death since knock out mutant is tolerant	[67]
*RCD1*	AT1G32230	RADICAL-INDUCED CELL DEATH1	mutants are sensitive to ozone and apoplastic superoxides but tolerant to ROS and UV-B stress	[67, 68]

**Table 4:** List of genes involved in plant DNA repair mechanism, cell cycle regulation and programmed cell death (PCD) in response to UV radiation.

Symbol	AGI code	Full name	Phenotype of UV-grown mutant seedlings	Reference
*UVR2/PHR1*	AT1G12370	UV REPAIR DEFECTIVE 2/ PHOTOLYASE 1	mutant is impaired in the CPD photolyase gene *PHR1* and thus, hypersensitive to high doses of UV-B	[82]
*UVR3*	AT3G15620	UV REPAIR DEFECTIVE 3	mutant is hypersensitive to high doses of UV-B as it is defective in photoreactivation of 6-4 photoproducts	[75]
*UVR1/UVH1*	AT3G28030	UV REPAIR DEFECTIVE 1	mutants are defective in dark repair or Nucleotide Excision Repair and shows hypersensitive to UV radiation	[72]
*CEN2*	AT4G37010	CENTRIN 2	mutants exhibited a moderate UV-C sensitivity due to defective Homologous Recombination and Nucleotide Excision Repair	[83]
*ATR*	AT5G40820	ATAXIA TELANGIECTASIA-MUTATED AND RAD3-RELATED	mutants is defective in cell-cycle arrest in response to DNA damage, and this causes elevated cell death under high level of UV-B	[80]
*ATM*	AT3G48190	ATAXIA-TELANGIECTASIA MUTATED	mutants show elevated cell death under high level UV-B due to DNA damage	[84]
*SOG1*	AT1G25580	SUPPRESSOR OF GAMMA RADIATION 1	mutant show reduced PCD in response to UV-B induced DNA photoproducts	[84]
*SUV2*	AT5G45610	SENSITIVE TO UV 2	mutant is UV-B sensitive which has a weaker expression of *CYCB1;1* in response to DNA damage	[85]
